# Effects of Resistant Starch on Symptoms, Fecal Markers, and Gut Microbiota in Parkinson’s Disease — The RESISTA-PD Trial

**DOI:** 10.1016/j.gpb.2021.08.009

**Published:** 2021-11-25

**Authors:** Anouck Becker, Georges Pierre Schmartz, Laura Gröger, Nadja Grammes, Valentina Galata, Hannah Philippeit, Jacqueline Weiland, Nicole Ludwig, Eckart Meese, Sascha Tierling, Jörn Walter, Andreas Schwiertz, Jörg Spiegel, Gudrun Wagenpfeil, Klaus Faßbender, Andreas Keller, Marcus M. Unger

**Affiliations:** 1 Department of Neurology, Saarland University, D-66421 Homburg, Germany; 2 Chair for Clinical Bioinformatics, Saarland University, D-66123 Saarbrücken, Germany; 3 Department of Human Genetics, Saarland University, D-66421 Homburg, Germany; 4 Department of Genetics/Epigenetics, Saarland University, D-66123 Saarbrücken, Germany; 5 Institute of Microecology, D-35745 Herborn, Germany; 6 Institute of Medical Biometry, Epidemiology and Medical Informatics, Saarland University, D-66421 Homburg, Germany; 7 Department of Neurology, Stanford University, Palo Alto, CA 94305, USA

**Keywords:** Parkinson’s disease, Short-chain fatty acid, Microbiota, Metagenomics, Intestinal inflammation

## Abstract

The composition of the gut microbiota is linked to multiple diseases, including Parkinson’s disease (PD). Abundance of bacteria producing **short-chain fatty acids** (SCFAs) and fecal SCFA concentrations are reduced in PD. SCFAs exert various beneficial functions in humans. In the interventional, monocentric, open-label clinical trial “Effects of Resistant Starch on Bowel Habits, Short Chain Fatty Acids and Gut **Microbiota** in **Parkinson’sDisease**” (RESISTA-PD; ID: NCT02784145), we aimed at altering fecal SCFAs by an 8-week prebiotic intervention with resistant starch (RS). We enrolled 87 subjects in three study-arms: 32 PD patients received RS (PD + RS), 30 control subjects received RS, and 25 PD patients received solely dietary instructions. We performed paired-end 100 bp length metagenomic sequencing of fecal samples using the BGISEQ platform at an average of 9.9 GB. RS was well-tolerated. In the PD + RS group, fecal butyrate concentrations increased significantly, and fecal calprotectin concentrations dropped significantly after 8 weeks of RS intervention. Clinically, we observed a reduction in non-motor symptom load in the PD + RS group. The reference-based analysis of metagenomes highlighted stable alpha-diversity and beta-diversity across the three groups, including bacteria producing SCFAs. Reference-free analysis suggested punctual, yet pronounced differences in the metagenomic signature in the PD + RS group. RESISTA-PD highlights that a prebiotic treatment with RS is safe and well-tolerated in PD. The stable alpha-diversity and beta-diversity alongside altered fecal butyrate and calprotectin concentrations call for long-term studies, also investigating whether RS is able to modify the clinical course of PD.

## Introduction

Gut microbiota composition is altered in Parkinson’s disease (PD) [1], [2], [3]. An increased abundance of Enterobacteriaceae has been consistently described in the fecal samples of PD patients, whereas the abundance of *Prevotella*, *Faecalibacterium*, *Blautia*, and *Bifidobacterium* is reduced in PD [1], [4], [5], [6], [7], [8]. This is of potential relevance since bacteria with anti-inflammatory properties (*e.g.*, synthesis of short-chain fatty acids, SCFAs) are less abundant in PD. Potentially pro-inflammatory bacteria (*e.g.*, endotoxin-containing species) are more abundant in PD. Members of the families Prevotellacae, Ruminococcacae, and Bacteroidacae are capable of fermenting resistant starch (RS), a nutritional component that arrives in the large intestine without previous degradation by human enzymes [9]. Anaerobic fermentation of RS results in SCFAs, such as butyrate [10]. Butyrate exerts essential functions in the gut: it represents the main energy source for enterocytes, enhances gut motility, and exerts immunomodulatory effects [9], [10]. Animal studies have shown that butyrate interacts with colonic regulatory T cells, creating an anti-inflammatory environment [11]. Consequently, a lack of SCFA-producing bacteria and reduced colonic SCFA concentrations presumably lead to reduced gut motility as well as to a shift in the intestinal immune system toward a more pro-inflammatory environment [12]. Intestinal inflammation, as well as altered gut motility (*e.g.*, constipation), has frequently been described in PD. In addition, we have previously shown that PD patients have reduced fecal SCFA concentrations compared to matched controls [6].

With regard to techniques used to characterize the microbiome, 16S amplicon sequencing has been most frequently used in microbiome studies due to its broad availability, moderate costs, and straightforward analysis. In recent years, whole-genome sequencing (WGS) has become widely available. Compared to 16S amplicon sequencing, WGS requires more complex computational and analytical procedures but is superior in characterizing the metagenomic landscape with regard to resolution, accuracy, and functional profiling [13], [14]. To characterize the metagenomic landscape, two different approaches can be used: 1) reference-free approaches characterize the metagenomic landscape based solely on sequencing data; 2) reference-based approaches rely on existing databases to compare the generated sequences against. In the present study, we computed the taxonomic profile with reference-based approaches. In addition, we also performed a comparative analysis with a hybrid approach named BusyBee [15]. BusyBee is a software combining both reference-free and reference-based approaches.

A sensitive and valid marker of intestinal inflammation is fecal calprotectin. Calprotectin is a protein in human leukocytes. In case of inflammation, leukocytes migrate into the intestinal lumen, and calprotectin can be measured in the feces as a stable marker that reflects even subclinical intestinal inflammation [16]. In accordance with the finding of prevailing pro-inflammatory bacteria in PD, elevated fecal calprotectin concentrations have been described in PD, too [17], [18].

A prebiotic approach to increase SCFA concentrations is nutritional supplementation with RS. The efficacy and tolerability of a 12-week intervention with RS have already been shown in a controlled clinical trial for elderly subjects (≥ 70 years old): RS was well-tolerated and, compared with placebo, elderly subjects on RS showed an altered intestinal microbiota, an increase in fecal butyrate concentrations, and a significant reduction in the use of laxatives [19].

Taken together, we set up the following hypothesis concerning a sequence of events: oral supplementation with RS enhances SCFA synthesis in the gut, probably accompanied by a shift in gut microbiota composition (due to a survival advantage for bacteria capable of fermenting RS). Consequently, the increased SCFA concentrations should lead to improved gut motility (improved constipation, respectively) and a reduction in markers of intestinal inflammation.

## Results

### The RESISTA-PD study cohort

**Figure 1:**
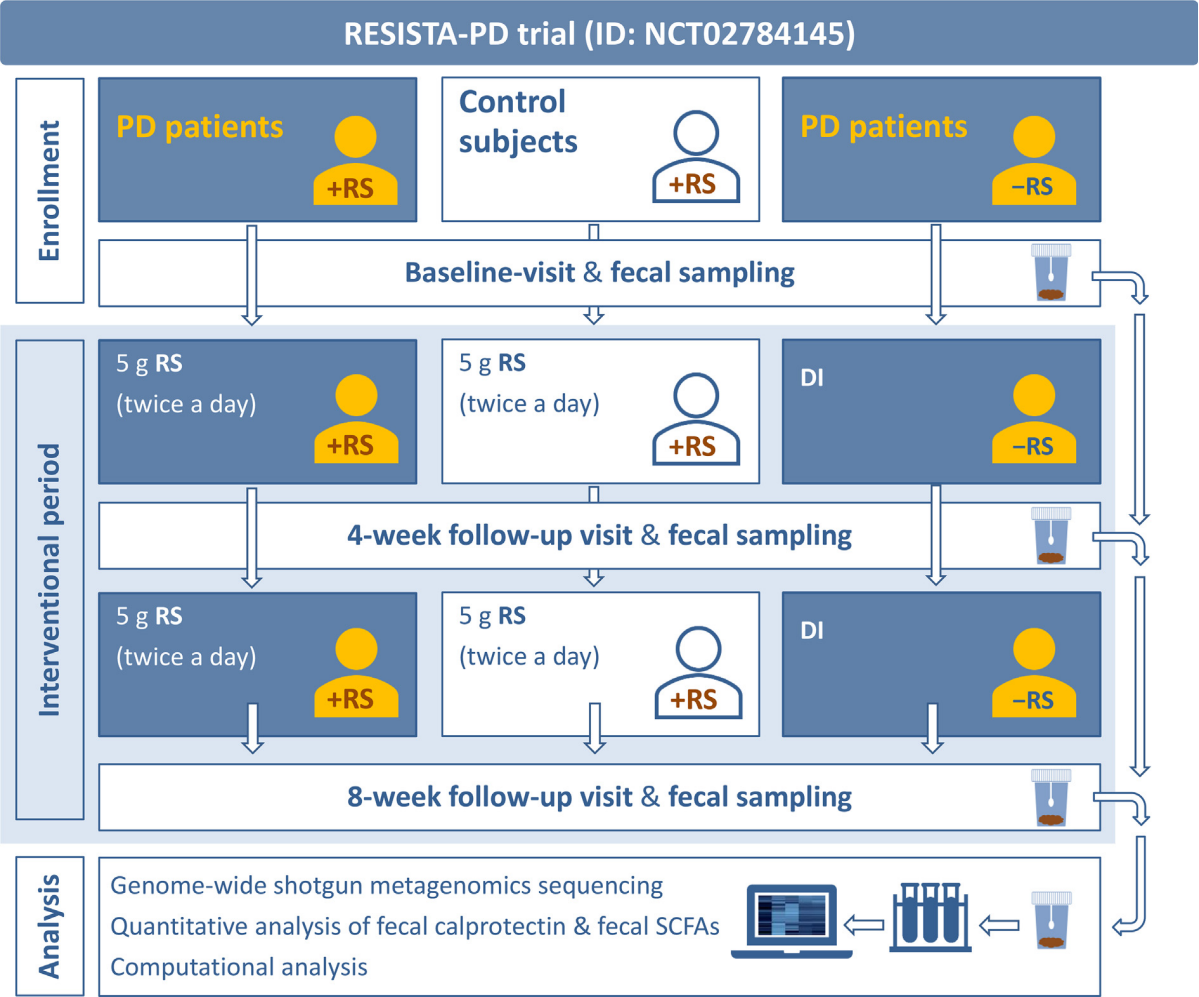
**Study design** Subjects were assigned to three different study-arms. One group of PD patients and a control group received 5 g RS twice a day for a total period of 8 weeks. The second group of PD patients solely received DI. Fecal samples and clinical scores were collected at baseline, after 4 weeks, and after 8 weeks for analysis. +RS in the pictograms visualizes subjects receiving RS, −RS in the pictograms visualizes subjects not receiving RS. PD, Parkinson’s disease; RS, resistant starch; SCFA, short-chain fatty acid; DI, dietary instruction.

Eighty-seven subjects participated in the trial “Effects of Resistant Starch on Bowel Habits, Short Chain Fatty Acids and Gut Microbiota in Parkinson’s Disease” (RESISTA-PD). The study design and workflow illustrating subjects’ allocation to study-arms, clinical visits, sample collection, and analysis are summarized in Figure 1. The majority of subjects (n = 76) completed the study per protocol. The median age was 64.5 years old in the PD group receiving RS (PD + RS), 66 years old in the PD group receiving dietary instruction (PD + DI), and 61.5 years old in the control group receiving RS (Co + RS). There was no significant difference regarding sex ratio between the groups. The majority of subjects were on an omnivorous diet. Additional epidemiologic and clinical data are summarized in Table 1, and detailed information regarding the medication of the enrolled subjects is provided in Table S1. No major side effects were reported during the 8-week intervention with RS.

**Table 1:** **Epidemiological and clinical characteristics of the enrolled subjects**

	**PD + RS**	**Co + RS**	**PD + DI**
Number of subjects enrolled	32	30	25
Reasons to withdraw from study by subject’s request after having started intervention	Subject disliked taste of RS (n = 1)	Acute disease of family member (n = 1)	Difficulties in handling fecal sampling kits at home (n = 1)
	General discomfort and upset stomach after starting RS (n = 3)		Claiming “personal problems and family issues” (n = 1)
	Concurrent acute disease after baseline visit (n = 1)		
Number of subjects with 4-week follow-up	28	29	23
Number of subjects with 8-week follow-up	26	27	23
Age (median, [range])	64.5 [42–84]	61.5 [40–76]	66 [47–80]
Sex (male / female)	18 / 14	12 / 18	13 / 12
Dietary habit	Omnivorous diet: n = 29	Omnivorous diet: n = 27	Omnivorius diet: n = 25
	Vegetarian diet: n = 2	Vegetarian diet: n = 2	Vegetarian diet: n = 0
	Pescetarian diet: n = 1	Pesceatrian diet: n = 1	Pescetarian diet: n = 0
Smoker	2 of 32	3 of 30	1 of 25
Disease duration in months (median, [range])	111 [7–288]	Not applicable	111 [22–265]
History of appendectomy	15 of 32	7 of 30	13 of 25
UPDRS I, II, III total score in on state (median, [range])	35 [4–74]	Not applicable	30 [3–69]
MMST (median, [range])	29 [23–30]	30 [28–30]	29 [25–30]

*Note*: PD + RS indicates PD patients receiving RS; Co + RS indicates control subjects receiving RS; PD + DI indicates PD patients receiving DI. PD, Parkinson’s disease; RS, resistant starch; DI, dietary instruction; UPDRS, Unified Parkinson’s Disease Rating Scale; MMST, Mini-Mental-Status-Test.

### Gut microbiota composition differs between PD patients and controls at baseline

**Figure 2:**
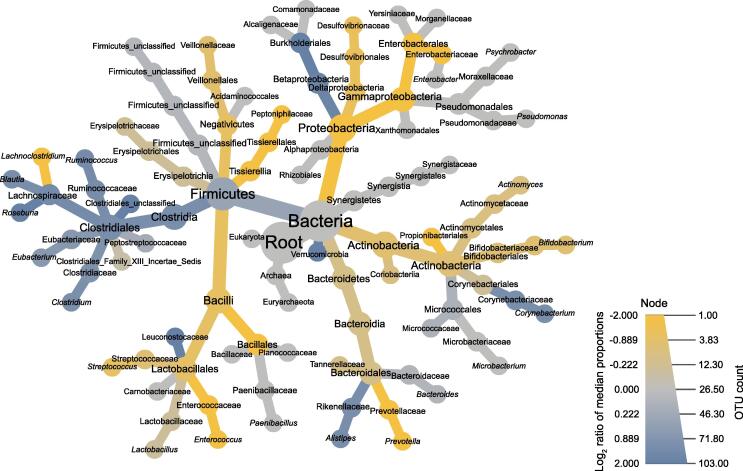
**Taxonomic tree illustrating differences between PD patients and controls at baseline** This taxonomic tree illustrates the number of OTUs per taxon (visualized by the size of the radius) and the difference (visualized by color) between PD patients and controls prior to intervention (baseline). Yellow shades indicate a higher abundance in PD patients; blue shades indicate a higher abundance in controls; gray shades indicate no group-specific differences. Low abundant taxa were pruned [46]. OTU, operational taxonomic unit.

At baseline, PD patients (n = 57) and controls (n = 30) showed no significant difference with regard to alpha-diversity with neither of the two applied analytical tools (MetaPhlAn2 and mOTUs2) (Figure S1A and B; File S1). With regard to beta-diversity, we observed a significant difference between PD patients and controls (*P* = 0.001) with both analytical tools applied in this study (Figure S1C and D). With regard to specific taxa*,* Lachnospiraceae *incertae sedis* (mOTU_v25_12240, *P* = 0.017) and *Faecalibacterium prausnitzii* (mOTU_v25_06110, *P* = 0.019) showed significantly reduced abundances after correction for multiple testing in PD patients compared to controls (Table S2). Figure 2 illustrates descriptive differences at different taxonomic levels between PD patients and controls prior to the intervention. Descriptively, taxa of the phylum Firmicutes showed higher abundances in controls (except for the class Bacilli), while most taxa of the phylum Proteobacteria, especially Enterobacteriaceae, were more abundant in PD.

### Intervention with RS alters symptom load and fecal markers in PD

We next analyzed the intervention-associated changes in subject-reported symptoms and in fecal markers. We observed a significant improvement with regard to non-motor symptoms [measured by the Non-Motor Symptoms Questionnaire (NMSQ) score, *P* = 0.001] and a significant improvement with regard to depressive symptoms [assessed by the Beck Depression Inventory (BDI), *P* = 0.001] in the PD + RS group at 8 weeks post intervention compared to the baseline (Table 2; Figure S2A). No significant changes in these parameters were identified over the 8-week intervention period for the PD + DI or Co + RS group. There was no significant change in bowel habits [assessed with the Constipation Scoring System (CSS)] between baseline and 8 weeks post intervention for any of the three investigated groups (Table 2; Figure S2A). Calprotectin concentrations dropped significantly in the PD + RS group at 8 weeks post intervention compared to the baseline (*P* = 0.023; Table 3; Figure S2B). No significant changes in fecal calprotectin concentrations were observed between baseline and 8 weeks post intervention in the Co + RS and PD + DI groups. Concerning fecal SCFAs, the concentration of the SCFA butyrate increased significantly in the PD + RS group at 8 weeks intervention compared to the baseline, for absolute fecal butyrate concentrations (*P* = 0.029) as well as for relative fecal butyrate concentrations (*P* = 0.026), (Table 4; Figure S2C); however, there were no significant changes for the concentrations of other SCFAs (including acetate, propionate, valerate, isobutyrate, and isovalerate) between baseline and 8 weeks post intervention in the PD + RS group (Table 4). Moreover, no significant changes in SCFA concentrations were observed between baseline and 8 weeks post intervention in the Co + RS and PD + DI groups (Table 4).

**Table 2:** **Scores on clinical scales at baseline and 8 weeks post intervention**

	**PD + RS**			**Co + RS**			**PD + DI**		
	**Baseline**	**Post intervention**	***P* value**	**Baseline**	**Post intervention**	***P* value**	**Baseline**	**Post intervention**	***P* value**
CSS score (median [range])	5 [0–14]	3.5 [0–15]	0.257	1 [0–11]	0 [0–8]	0.125	2 [0–10]	2 [0–12]	0.674
NMSQ score (median [range])	10.5 [3–20]	7.5 [2–18]	0.001	3 [0–9]	3 [0–10]	0.774	10 [4–19]	10 [5–19]	0.152
BDI score (median [range])	6.5 [2–25]	3 [0–12]	0.001	2 [0–14]	2 [0–20]	0.202	7 [1–18]	6 [0–13]	0.106

*Note*: CSS, Constipation Scoring System; NMSQ, Non-Motor Symptoms Questionnaire; BDI, Beck Depression Inventory.

**Table 3:** **Fecal calprotectin concentrations at baseline and 8 weeks post intervention**

	**PD + RS**			**Co + RS**			**PD + DI**		
	**Baseline**	**Post intervention**	***P* value**	**Baseline**	**Post intervention**	***P* value**	**Baseline**	**Post intervention**	***P* value**
Concentration of fecal calprotectin (median [range], µg/g feces)	56.8 [19–327]	20.5 [19–407]	0.023	19 [19–69]	19 [19–155]	1.000	46 [19–219]	31 [19–217]	0.481

**Table 4:** **SCFA concentrations at baseline and 8 weeks post intervention**

	**PD + RS**			**Co + RS**			**PD + DI**		
	**Baseline**	**Post intervention**	***P* value**	**Baseline**	**Post intervention**	***P* value**	**Baseline**	**Post intervention**	***P* value**
Butyrate (median [range], mmol/g)	0.24 [0.00–1.00]	0.25 [0.01–1.92]	0.029	0.46 [0.04–2.10]	0.32 [0.07–2.45]	0.223	0.29 [0.05–1.44]	0.27 [0.06–2.38]	0.426
Butyrate (median [range], %)	10.5 [0.9–22.2]	11.3 [0.9–21.4]	0.026	12.8 [4.5–21.1]	11.2 [4.8–22.5]	0.260	11.3 [3.9–18.8]	10.3 [2.1–20.6]	0.685
Acetate (median [range], mmol/g)	1.39 [0.15–4.91]	1.80 [0.23–6.55]	0.165	1.99 [0.57–6.78]	2.04 [0.38–9.54]	0.891	2.17 [0.24–36.0]	2.04 [0.65–7.66]	0.685
Propionate (median [range], mmol/g)	0.36 [0.03–1.74]	0.55 [0.06–2.22]	0.145	0.48 [0.08–1.50]	0.49 [0.12–2.16]	0.785	0.53 [0.11–1.66]	0.48 [0.18–1.94]	0.445
Valerate (median [range], mmol/g)	0.07 [0.01–0.29]	0.07 [0.02–0.18]	0.647	0.06 [0.02–0.26]	0.07 [0.02–0.20]	0.703	0.11 [0.01–0.35]	0.07 [0.01–0.66]	0.733
Isobutyrate (median [range], mmol/g)	0.05 [0.01–0.26]	0.05 [0.01–0.15]	0.829	0.05 [0.02–0.19]	0.05 [0.02–0.16]	0.715	0.07 [0.01–0.24]	0.05 [0.01–0.45]	0.501
Isovalerate (median [range], mmol/g)	0.04 [0.00–0.18]	0.04 [0.00–0.19]	0.461	0.06 [0.00–0.20]	0.04 [0.00–0.22]	0.584	0.06 [0.00–0.26]	0.04 [0.01–0.39]	0.537
Total SCFA (median [range], mmol/g)	2.08 [0.21–8.02]	2.72 [0.42–10.6]	0.142	3.17 [0.76–10.5]	2.96 [0.64–14.6]	0.973	3.37 [0.47–36.3]	3.07 [0.97–13.2]	0.745

*Note*: *P* values refer to the change between baseline concentrations and post-interventional concentrations. SCFA, short-chain fatty acid.

### Reference-based analysis shows a stable gut microbiome after RS intervention

**Figure 3:**
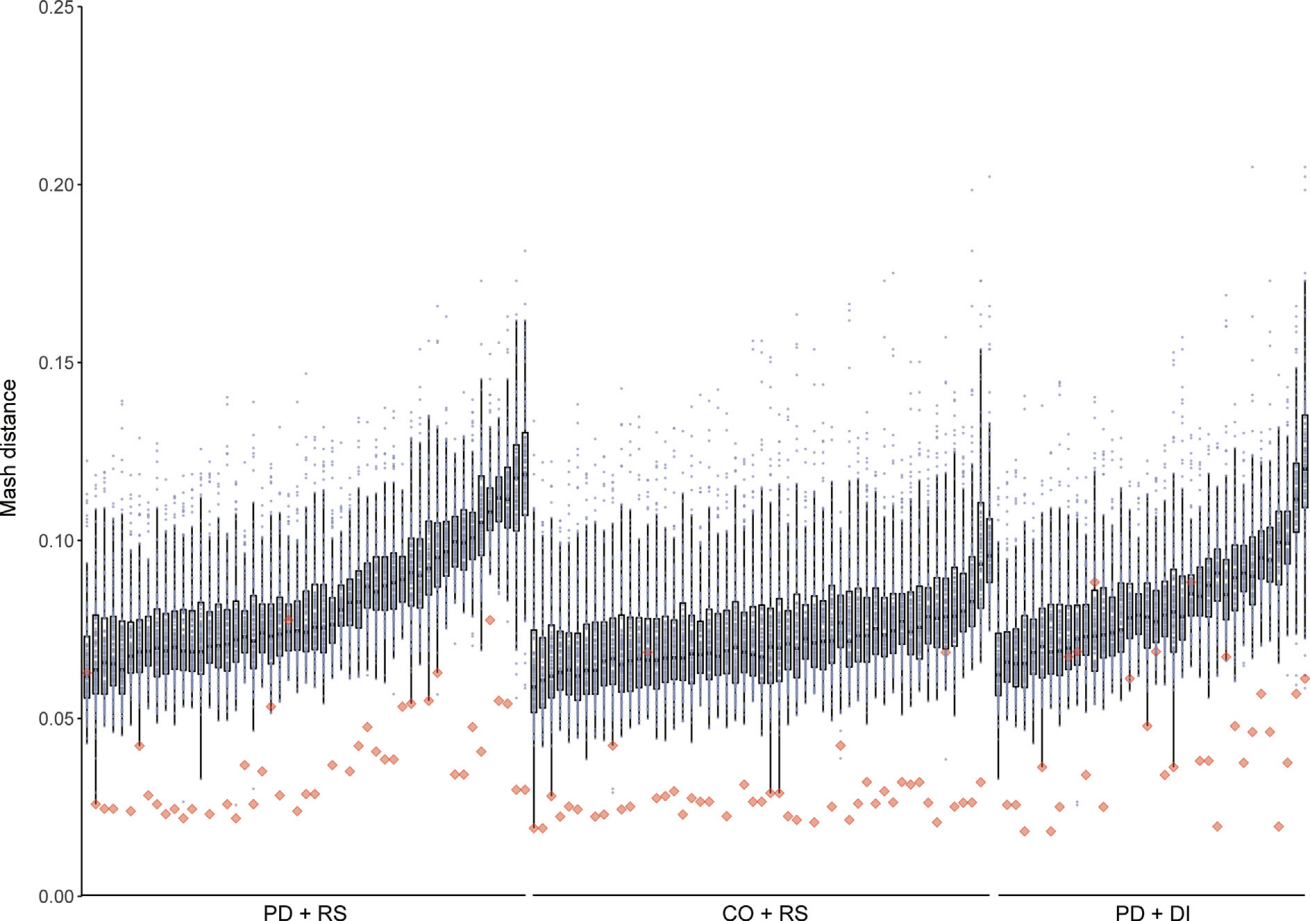
**High intra-individual and low inter-individual similaritiesof samples** The similarity of samples visualized as Mash distance plot (grouped by study-arms). The lower the Mash distance, the higher the similarity of samples. Red diamonds represent paired samples (baseline and 8 weeks post intervention) of one subject. Dots represent samples of other subjects (unpaired).

In order to investigate whether the observed changes in clinical symptoms and fecal markers are associated with an intervention-associated shift in the gut microbiome, we performed metagenomic sequencing. Quality control by FastQC indicated good data quality of metagenomic sequencing. During preprocessing, less than 1% of reads were removed for each sample. In addition to the standard quality control, we analyzed pairwise Mash distances [20] between all samples. Hereby, the Mash distance gauges similarity between sequencing libraries using the only sequence features directly derived from raw reads. Visualizing Mash distances showed that samples derived from the same individual frequently produced the lowest Mash distance, indicating correct labeling of samples and a lack of contamination (Figure 3). No significant intervention-associated changes with regard to either alpha-diversity or beta-diversity were detected for any of the three investigated groups (PD + RS, PD + DI, and Co + RS). No significant intervention-associated changes were detected concerning differences in distinct taxa (Table S2). Non-metric multidimensional scaling (NMDS) visualizing microbiome shifts did not reveal uniform shifts associated with the intervention (Figure S3).

### Reference-free analysis points at punctual differences in the metagenomic signature

**Figure 4:**
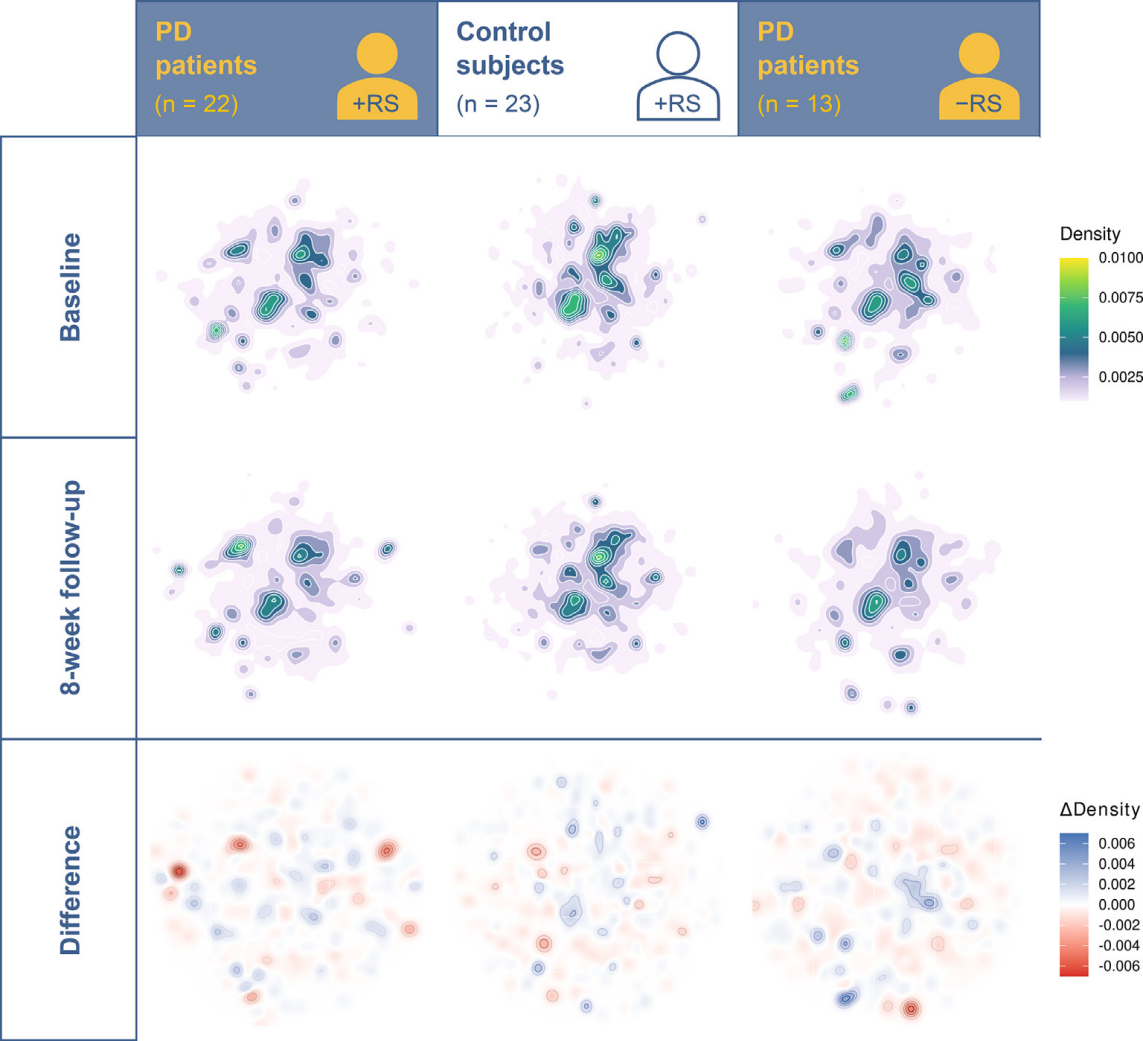
**Reference-free analysis points at punctual differences** This figure shows the density distribution of the 5-mers, after dimensionality reduction with UMAP. The first row contains the baseline. The second row shows the 8-week follow-up. In the bottom row, the difference between the two previous rows is visualized; blue indicates a stronger signal at baseline, and red indicates a stronger signal at 8 weeks follow-up. UMAP, uniform manifold approximation and projection.

Reference-free analysis revealed intervention-associated changes in taxonomic signatures in the PD + RS group (Figure 4). The majority (> 54%) of contigs forming one of the three clusters in the reference-free analysis were derived from the genus *Rhodococcus* (Figure S4). Density changes worth interpreting as clusters identified in the other cohorts (Co + RS and PD + DI) did not contain significant amounts of *Rhodococcus* sequences.

### Distinct microbial signatures are associated with fecal butyrate concentrations

**Figure 5:**
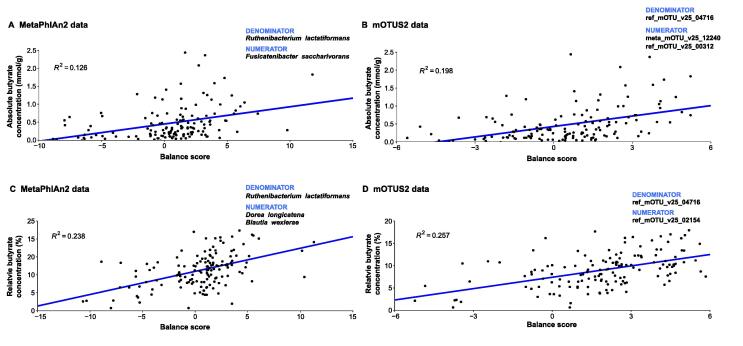
**Distinct microbial signatures are associated with fecal butyrate concentrations** **A.** and **B.** Balance scores for MetaPhlAn2 data (A) and mOTUS2 data (B) with absolute butyrate concentrations as response variables. **C.** and **D.** Balance scores for MetaPhlAn2 data (C) and mOTUS2 data (D) with relative butyrate concentrations as response variables.

In metagenomic samples, the change in the abundance of one taxon is likely to entail changes in the abundance of other taxa. We investigated our data for data compositionality using the selbal algorithm. Selbal searches for two groups of taxa whose relation (or balance) is associated with a certain response variable. The relationship is modeled as a linear or logistic regression model of the taxa on the response variable. Selbal builds multiple models containing different taxa combinations and evaluates their performances using cross-validation. In our dataset, response variables were measurements of acetate, propionate, butyrate, valerate, and calprotectin, as well as CSS and BDI scores. Using the selbal algorithm, results for MetaPhlAn2 and mOTUs2 data (Figure 5) with butyrate concentrations as response variables were highly consistent. For absolute butyrate concentrations, selbal detected that higher abundances of *Fusicatenibacter saccharivorans* to *Ruthenibacterium lactatiformans* were associated with higher absolute butyrate concentrations (Figure 5A) with an association slightly below moderate (MetaPhlAn2 data, *R*^2^ = 0.126). The association of *Ruthenibacterium lactatiformans* with butyrate concentrations was verified by mOTUs2 data. Here, selbal detected that higher abundances of Lachnospiraceae and *Streptococcus parasanguinis* to *Ruthenibacterium lactatiformans* were associated with higher absolute butyrate concentrations, and the association was moderate (*R*^2^ = 0.198; Figure 5B). For relative butyrate concentrations, selbal detected that higher abundances of *Dorea longicatena* (MetaPhlAn2 data) and *Blautia wexlerae* (MetaPhlAn2 and mOTUs2 data) to *Ruthenibacterium lactatiformans* (MetaPhlAn2 and mOTUs2 data) were associated with higher relative butyrate concentrations, and the association was moderate (*R*^2^ = 0.238 for MetaPhlAn2 data, *R*^2^ = 0.257 for mOTUs2 data; Figure 5C and D). The model itself was stable, with *Ruthenibacterium lactatiformans* and *Dorea longicatena* being included in over 95% of all models for MetaPhlAn2 data and *Blautia wexlerae* and *Ruthenibacterium lactatiformans* being included in over 96% of all models for mOTUs2 data. Other response variables did not show consistency between mOTUs2 and MetaPhlAn2 data.

### Functional profiling reveals no intervention-associated difference in metabolic pathways

In order to identify differences in the available metabolic pathways, we applied the HUMAnN2 tool to our data. The estimated pathway abundances were used for an exploratory data analysis of the samples using principal component analysis (PCA) and a differential analysis using ALDEx2. The PCA projection indicated a different tendency between the PD + RS and Co + RS groups, but no differences associated with the intervention (baseline *vs.* 8 weeks post intervention) (Figure S5). The analysis with ALDEx2 did not result in any pathway that showed a significant difference between groups nor a difference between baseline and 8 weeks post intervention (Table S3).

## Discussion

Gut microbiota composition is altered in PD [3], [4], [5], [6] and might be a contributing factor for gastrointestinal non-motor symptoms (*e.g.*, constipation) in PD. Having recognized the relevance of the intestinal microbiome in PD, probiotics have been investigated in PD and other neurodegenerative diseases previously [21], [22] and prompted us to perform the RESISTA-PD trial.

In accordance with other studies in the field [2], [4], [5], [7], [23], [24], we observed a difference between PD patients and controls at baseline regarding beta-diversity. With regard to specific taxa, we detected significantly different abundances for two taxa after correction for multiple testing: abundances for Lachnospiraceae *incertae sedis* and *Faecalibacterium prausnitzii* were significantly reduced in the fecal samples of PD patients. Lachnospiraceae as well as *Faecalibacterium prausnitzii* have already been reported to be reduced in PD and have also been confirmed as altered taxa in PD in a recent meta-analysis [25]. Indeed, the lower abundance of *Faecalibacterium prausnitzii* might be one explanation for the lower fecal butyrate concentrations in PD. On a descriptive level, we also reproduced some other previously reported alterations of the gut microbiota in PD, *e.g.*, a lower abundance of Firmicutes and a higher abundance of Proteobacteria, especially Enterobacteriaceae.

For the reference-free analysis of intervention-associated changes, a metagenomic signature indicating a possible involvement of *Rhodococcus* was found in the PD + RS group, despite an insignificant change in abundance during the read-based analysis. As shown in Figure 4, the blue cluster (top right) in the CO + RS group contained only sequences derived from one single sample, and the sequences were assigned to *Rhodotorula toruloides*; the two clusters with the highest density in the PD + DI group contained no sequences of the genus *Rhodococcus* (blue cluster) and less than 0.001% of sequences of the genus *Rhodococcus* (red cluster). The reference-free workflow we selected discards all quantity information after assembly. In an optimal scenario, sequences derived from identical genetic information will be collapsed into the same contig in each sample. In BusyBee, one such contig will appear as one individual point with close to no impact on the overall density distribution. The strong signal from the high-density cluster in the PD + RS group suggests the existence of multiple contigs that are dissimilar enough not to be collapsed during assembly yet qualitatively good enough to be assigned to *Rhodococcus*. Accordingly, the change in the density of the investigated cluster indicates a more complex behavior than a quantitative balance shift. Instead, an increase in genomic diversity may be postulated from this observation. The relevance of this particular finding remains unclear and requires further investigations, especially since the genus *Rhodococcus* is not a typical representative of the human gut microbiota. Given the fact that bacteria of the genus *Rhodococcus* are not typical part of the human gut microbiota and also are obligate aerobes, the identification of this genus in human fecal samples points at potential contamination. Indeed, *Rhodococcus* has been identified in human biosamples due to DNA contamination of reagents [26]. Yet, DNA contamination is mainly a problem when analyzing low microbial biomass samples (like blood or saliva). In our study that analyzed high microbial biomass samples (feces), contamination is further unlikely as contamination would have occurred solely in PD + RS samples after the intervention and not in the other two groups. One might also hypothetically consider contamination of a single batch of reagents or tubes used in our study. However, samples were analyzed in a random way and not sorted by the group prior to analysis. Hence, contamination can hardly explain this finding. As the taxonomic assignment of contigs relies on libraries, misassignment due to similar sequences of another taxon (not represented in libraries) with sequences of *Rhodococcus* should also be considered.

Our finding that a prebiotic intervention with RS significantly alters fecal butyrate concentrations and significantly reduces fecal calprotectin concentrations is also in line with a controlled clinical trial that investigated RS in mid-age and elderly subjects and reported an increase in fecal butyrate concentrations in subjects aged 70 years or older [19]. While the study by Alfa and colleagues [19] even indicated a therapeutic effect (reduction in the use of laxatives), we did not observe a significant improvement of bowel habits. This divergent observation between our study and the study by Alfa and colleagues might be due to the differences in the types of RS (we used RS type 3, while Alfa and colleagues used RS type 2), the doses of RS (Alfa and colleagues administered approximately double the dosage compared to our study), and the duration of the interventional period (8 weeks in our study *versus* 12 weeks in the study by Alfa and colleagues).

Fecal butyrate concentrations and calprotectin concentrations were not altered when PD patients solely underwent nutritional counseling, including DI concerning a fiber-rich diet (PD + DI). Considering the fact that the PD + DI group underwent the same visit schedule as the PD + RS group, the effect observed with regard to clinical measures in the PD + RS group is unlikely to be completed due to unspecific effects such as attention paid to subjects during clinical visits or answering questionnaires according to social desirability.

The effect on symptoms related to depression in the PD + RS group might be explained by the observed increase in butyrate concentrations. An association between gut microbiota and depressive symptoms has been described previously [27], [28]. Administration of SCFAs, including butyrate, has been shown to reduce depressive symptoms in mice [29]. Moreover, fecal SCFAs have been shown to be reduced in a cohort of female patients with depression [30]. Increasing evidence suggests a connection between depressive symptoms and fecal SCFA concentrations [31]. One explanation for the lack of a change in clinical measures in the PD + DI group might be that adherence to DI is likely to be lower compared to the more convenient approach of consuming a dietary supplement (dissolved in one glass of water) twice a day. In addition, changes in dietary habits are much more heterogeneous compared to standardized nutritional supplementation.

The fact that the Co + RS group did not show a reduction in fecal calprotectin concentrations is likely to be explained by already normal calprotectin concentrations in control subjects at baseline. The unchanged SCFA concentrations in the Co + RS group might be either explained by a ceiling effect or by a lower adherence (as controls did not expect to benefit from the intervention).

Even though the effects on fecal calprotectin and fecal butyrate were significant in the PD + RS group and also SCFAs other than butyrate showed a trend towards an increase in concentrations in the PD + RS group, our data lack a clear-cut correlate concerning specific gut microbiota. Assuming that gut microbiota composition remains stable despite the prebiotic intervention, an altered transcription might have led to the observed effects on fecal markers. The lack of a clear-cut response to the intervention with regard to gut microbiota or symptoms of constipation might also be due to various individual factors. Our study design controlled for confounding factors like age, sex, overall type of diet, comorbidities, and medication. Nevertheless, the investigated cohorts were heterogeneous (even within groups) with regard to other, more complex factors that might determine the individual response (*e.g.*, the composition of the gut microbiome prior to the intervention, adherence to the recommended RS intake, more specific dietary habits). This said, the limited sample size in this proof of concept study together with the inter-individual variability concerning potential confounding factors, is one explanation for the heterogeneous response to the intervention. Hence, larger cohorts (as well as transcriptomics and proteomics) might have been necessary to detect more subtle intervention-associated alterations in the gut microbiome (and possible changes at the transcriptomic level).

In order to identify microbial signatures associated with SCFA concentrations, we performed an *in silico* analysis (using the selbal algorithm): balance analysis of taxa and butyrate concentrations resulted in concordant results for both analytical tools (mOTUs2 and MetaPhlAn2). Moreover, we confirmed the robustness of the identified balance scores by their frequency in a cross-validation model. The microbial taxa *Blautia wexlerae*, *D. longicatena*, and *Ruthenibacterium lactatiformans* are involved in butyrate-related pathways [32]. However, all of these bacteria are not capable of directly producing butyrate from RS, but they produce lactate and succinate by fermentation which consecutively serves as substrates for other bacteria which produce butyrate [10]. Despite the fact that our *in silico* approach did not detect classical SCFA producers (like *Faecalibacterium* or *Roseburia*) as determinants for fecal butyrate concentrations, the taxa identified by selbal are indirectly involved in butyrate production (via complex interactions with other taxa) [10].

In contrast to our initial hypothesis, symptoms related to constipation (a frequent non-motor symptom in PD) were not significantly altered during the 8-week intervention. As there was at least a descriptive decline in CSS scores after RS supplementation, we suggest longer interventional periods and increased doses of RS to test such a symptomatic effect on bowel habits. Given that RS was well-tolerated in the RESISTA-PD trial, this seems to be a feasible and rational approach.

Besides the limited sample size and the relatively short interventional period, one main limitation of the RESISTA-PD trial is its open-label study design. We aimed at counteracting this shortcoming by including an additional PD control-arm (PD + DI) to control for unspecific effects (as discussed above). Adherence to the intervention was checked by patient diaries but not by more objective measures. Probably, an internal motivation to adhere to the study protocol might have been higher in the PD + RS group compared to the Co + RS group (as discussed above). The primary aim of the RESISTA-PD trial was to test the feasibility, tolerability, and efficacy of this prebiotic approach. Hence, our study protocol did not include an additional measurement of the investigated markers several weeks after withdrawal of RS. We suggest including such an assessment in future studies.

At this time, we are not able to answer the question of whether the observed anti-inflammatory effects indicated by the decline in fecal calprotectin concentrations are mediated by the increase in butyrate concentrations. Even though other studies endorse such an assumption [11], further studies are needed to clarify the exact mechanisms of this prebiotic intervention and the increase in SCFAs in detail.

A general limitation of interventions aiming at altering the gut microbiome is the question of endurance. This is why long-term studies and assessment of subjects after withdrawal of the intervention are mandatory to draw final conclusions.

## Conclusion

RS, as a dietary supplement to increase fiber intake, is safe and well-tolerated in PD. RS supplementation partially restores fecal SCFA concentrations in the PD + RS group without clear-cut changes in the gut microbiome that were attributable to the intervention. Alterations at the transcriptome level that are not captured by our approach might explain the intervention-associated significant increase in fecal markers in the PD + RS group.

In view of the good tolerability of RS, we suggest long-term studies with RS. These studies should also aim at clarifying the underlying mechanisms for the supposed anti-inflammatory effects. Based on the assumption of an RS-associated anti-inflammatory effect, these studies should also investigate whether RS supplementation is able to modify the clinical course of PD.

## Materials and methods

### Study design and registration

The interventional study RESISTA-PD is a monocentric, prospective, open-label clinical trial investigating the effects of an 8-week prebiotic intervention with the dietary supplement RS (Catalog No. P/N 03647989, SymbioIntest, SymbioPharm GmbH, Herborn, Germany) (5 g RS twice a day orally) in PD patients (PD + RS) and matched controls (Co + RS). As a third study-arm, PD patients who received solely DI (PD + DI) were enrolled in this study. DI was based on the “Food-Based Dietary Guidelines in Germany” (for further reference, see https://www.dge-medienservice.de/food-based-dietary-guidelines-in-germany.html) of the German Nutrition Society. At the baseline visit, the specified guidelines to support a health-promoting diet were explained to all subjects in the PD + DI group. These recommendations support a diet rich in whole-grain products and vegetables and moderate consumption of fat and animal products. Subjects also received a leaflet summarizing these recommendations. This leaflet included a table with practical orientation values for each food group (*e.g.*, cereal products and potatoes, vegetable and salad, and fruit). Primary outcome measures were: change (prior- *vs.* post-intervention) in (a) bowel habits, (b) fecal SCFA concentrations, and (c) gut microbiome (analyzed by whole genome-wide sequencing). Secondary outcome parameters were: differences in gut microbiome at baseline (between PD patients and controls), change (prior- *vs.* post-intervention) in clinical scales, and change in fecal calprotectin concentrations (prior- *vs.* post-intervention).

### Subjects

A total of 57 PD patients and 30 control subjects were enrolled. PD patients were assigned to two different interventional groups: PD + RS (n = 32) received 5 g RS twice per day orally over a period of 8 weeks; PD + DI (n = 25) received DI concerning high fiber intake, but no RS supplementation. Control subjects (Co + RS, n = 30) received 5 g RS twice per day orally over a period of 8 weeks. The main inclusion criteria were an age > 18 years old, diagnosis of PD (respective absence of PD or any other neurodegenerative disorder in the control group), capacity to give written informed consent. The main exclusion criteria were use of antibiotics, steroids, antimycotics or probiotic supplements (during the last 12 weeks), chronic or acute disorders of the gastrointestinal tract (other than constipation), a history of colonoscopy within the past 12 weeks, a history of gastrointestinal surgery (other than appendectomy) within the past three years.

### Clinical assessments

Subjects were assessed at baseline, 4 weeks post intervention, and 8 weeks post intervention. Baseline assessment was performed as in-person clinical visit. Assessments for 4 weeks and 8 weeks post intervention respectively, were performed as telephone visits. At baseline visit, subjects underwent rating with the Unified Parkinson’s Disease Rating Scale (UPDRS) [33] and the Mini-Mental-Status-Test (MMST) [34]. Symptoms related to constipation were assessed at each of the three visits (baseline, 4 weeks post intervention, 8 weeks post intervention) with the CSS [35]. Depressive symptoms and non-motor symptoms were assessed with the BDI scores [36] and the NMSQ scores [37], respectively, at baseline and 8 weeks post intervention. In addition to collecting data on adverse events, tolerability and subjective improvement of the intervention were assessed in analogy to the seven-point Clinical Global Impression - Improvement (CGI-I) scale [38] after 4 and 8 weeks of intervention. The clinical change was rated (compared to baseline, prior to the intervention with RS respectively) as: very much improved, much improved, minimally improved, no change, minimally worse, much worse, or very much worse.

### Collection of fecal samples

At the baseline visit, all subjects received sterile containers (Calalog Nos. P/N S1000-150 and P/N H9550T, Suesse, Gudensberg, Germany) for the collection of fecal samples at home. The containers were labeled with the subject-ID and the scheduled time for collection (baseline, *i.e.*, prior to the first intake of RS; 4 weeks of intervention with RS; and 8 weeks of intervention with RS). All subjects were instructed how to collect the fecal samples at home and received a leaflet containing relevant information for sample collection. Subjects were instructed to send in two samples (collected on two consecutive days) for each time point. For metagenomic sequencing, the first baseline sample and the first 8-week sample were used. For quantitative analysis of fecal markers, the mean of the two samples was calculated for further statistical analysis. In case of missing 8-week samples, 4-week samples were analyzed (last observation carried forward, LOCF) as described below (see the “Statistical analysis of clinical data and fecal SCFA and calprotectin concentrations” section). All subjects were reminded by telephone to send in samples after 4 weeks and after 8 weeks of intervention. Stool samples were sent to the Institute of Microoecology, Herborn, Germany, and immediately frozen at −35 °C until analysis.

### Measurement of fecal SCFA and calprotectin concentrations

Quantitative analyses of fecal SCFAs and calprotectin were carried out by the Institute of Microoecology, Herborn, Germany. All persons involved in these analyses were blinded to clinical data and the diagnosis of the subjects. Fecal SCFAs were measured by gas chromatography; fecal calprotectin was measured by enzyme-linked immunosorbent assay as previously described [6], [18].

### DNA isolation

DNA from fecal samples was isolated using the DNeasy PowerSoil Kit (Catalog No. P/N 47014, QIAGEN, Hilden, Germany) according to the manufacturer’s instructions. To ameliorate the purity, we performed precipitation of the DNA in the presence of sodium acetate (pH = 5.5) and cold 100% ethanol at −20 °C for at least overnight. The DNA was then centrifuged, washed with 80% ethanol once, and centrifuged another time. The pellet was air-dried and resuspended in TE buffer. DNA concentration was measured using a Nanodrop 2000 spectrophotometer (Catalog No. P/N ND-2000, ThermoFisher Scientific, Waltham, MA).

### Metagenomic sequencing

DNA libraries were prepared using the MGIEasy DNA Library Prep Kit (Catalog No. P/N 940-200022-00, MGI Technologies, Shenzhen, China) according to the manufacturer's recommendations. In general, 1 µg of input DNA was sheared into fragments using the M220 Focused-ultrasonicator (Catalog No. P/N 500295, Covaris, Woburn, MA). Size selection was carried out using Agencourt AMPure XP beads (Catalog No. P/N A63882, Beckman Coulter, Krefeld, Germany). Then, 50 ng of fragmented DNA were used for end-repairing and A-tailing followed by ligation of barcode containing adaptors to the 3′- and 5′-ends. The ligation products were amplified by PCR. A total of 16 different barcoded samples were pooled in equal amounts and circularized using a specific oligo sequence, which is complementary to the sequences in the 3′- and 5′-adaptors. DNA nanoballs (DNBs) were generated by rolling circle amplification (RCA), and loaded onto a flowcell using BGIDL-50 DNB loader. Paired-end sequencing was performed according to the BGISEQ-500RS High-throughput Sequencing Set for PE100 on the BGISEQ-500RS instrument (Catalog No. P/N 940-100037-00, MGI Technologies).

### Statistical analysis of clinical data and fecal SCFA and calprotectin concentrations

In case of missing data for 8 weeks post intervention and available data for 4 weeks post intervention, we applied the LOCF method. LOCF was used for 4 subjects [PD + RS (n = 3) and PD + DI (n = 1)] to replace missing 8-week data concerning fecal markers. Concerning clinical scores, missing 8-week data of 5 subjects [PD + RS (n = 2), PD + DI (n = 1), and Co + RS (n = 2)] were replaced by 4-week data. The normal distribution of data was tested using the Shapiro-Wilk’s test. Statistical significance was assumed for *P* < 0.05. The difference between groups was tested using the Mann-Whitney-U-test. Comparisons of the same group at different time points were performed with the Wilcoxon’s test for paired samples and the sign test for paired samples. Pre-defined outcome measures were not adjusted for multiple testing. Spearman’s correlation coefficient was used to analyze correlations between parameters.

### Sequencing data analysis

#### Preprocessing

FastQC (version 0.11.8) was used to validate sequence quality, and the reports were summarized using multiQC (version 1.7) [39]. Adapter contamination was controlled with the Minion tool from the Kraken package (version 16.098) [40]. None of the samples showed adapter contamination. Trimming and host contamination removal were conducted using KneadData (version 0.7.2; https://huttenhower.sph.harvard.edu/kneaddata/, accessed 30 Aug 2020).

#### Read-based analysis

Taxonomic composition of the samples was profiled using mOTUs2 (version 2.5.0) [41] as well as MetaPhlAn2 (2.9.19) [42]. Both methods are marker-based and were used to profile all taxonomic levels. Functional profiling was conducted using HUMAnN2 (version 2.8.1) [43]. The R-package phyloseq (version 1.28.0) [44] was used to plot the relative abundances in each sample at different taxonomic levels, ranging from kingdom to species. Alpha-diversity was computed using multiple measurements for each sample. The distributions of the alpha-diversity values were compared between patient groups for the same time point and between time points for the same patient group. Beta-diversity was calculated using the Bray-Curtis distance. Differential abundance analysis was performed by comparing the taxa abundance between groups at the same timepoint and within groups for different time points using the R-package ALDEx2 (version 1.14.1) [45]. Metacoder R-package [46] was used to visualize differences in taxa abundance between PD patients and controls. Regression-based balance analysis of the taxa was done using the R-package selbal (version 0.1.0) [47]. For analysis with the selbal algorithm, we included all samples and all time points. Mash distances were computed on the preprocessed reads using Mash (version 2.1.1) [20].

#### Reference-free analysis

Reference-free analysis closely resembled the BusyBee workflow [15], which is centered around *k*-mers. *De novo* assembly was performed using SPAdes (version 3.13.1) [48] for all samples with matching baseline and 8-week follow-up datasets. The obtained contigs were filtered by length, and sequences shorter than 5000 bp were discarded. Of these filtered sequences longer than 5000 bp, 5-mers and reverse complement 5-mers distributions were computed. Samples were then pooled, and a uniform manifold approximation and projection (UMAP) was computed [49]. The embedded data points were then reassigned to their respective group-time point combination. Contigs for further analysis were taken from the PD + RS group lying within the UMAP coordinates 16.8 < X < 18.2 and 7.5 < Y < 11. The remaining contigs were analyzed with BusyBee. The reported taxonomic assignment of the filtered contigs was computed with CAT/BAT (version 5.0.3) [50].

## Ethical statement

The study was reviewed and approved by the ethics committee of the Medical Association of Saarland, Saarbruecken, Germany, and registered under the reference number 189/15. The study was registered at the clinical trials registry ClinicalTrials.gov (ID: NCT02784145). Written informed consent was obtained from all subjects prior to inclusion in the study.

## Data availability

Data obtained in this study have been deposited in the Genome Sequence Archive for Human [51] at the National Genomics Data Center, Beijing Institute of Genomics, Chinese Academy of Sciences / China National Center for Bioinformation (BioProject: PRJCA004435), and are publicly accessible at https://ngdc.cncb.ac.cn/gsa-human/.

## CRediT author statement

**Anouck Becker:** Conceptualization, Formal analysis, Investigation, Writing - review & editing, Visualization. **Georges Pierre Schmartz:** Methodology, Software, Validation, Formal analysis, Data curation, Writing - review & editing, Visualization. **Laura Gröger:** Methodology, Investigation, Writing - review & editing. **Nadja Grammes:** Methodology, Software, Validation, Formal analysis, Data curation, Writing - review & editing, Visualization. **Valentina Galata:** Methodology, Software, Validation, Formal analysis, Data curation, Writing - review & editing, Visualization. **Hannah Philippeit:** Investigation, Formal analysis, Writing - review & editing. **Jacqueline Weiland:** Investigation, Formal analysis, Writing - review & editing. **Nicole Ludwig:** Methodology, Investigation, Writing - review & editing. **Eckart Meese:** Methodology, Resources, Writing - review & editing, Supervision. **Sascha Tierling:** Investigation, Writing - review & editing. **Jörn Walter:** Methodology, Resources, Writing - review & editing, Supervision. **Andreas Schwiertz:** Investigation, Resources, Writing - review & editing. **Jörg Spiegel:** Investigation, Writing - review & editing. **Gudrun Wagenpfeil:** Formal analysis, Writing - review & editing. **Klaus Faßbender:** Conceptualization, Resources, Writing - review & editing. **Andreas Keller:** Conceptualization, Methodology, Software, Validation, Formal analysis, Resources, Data curation, Writing - review & editing, Visualization, Supervision. **Marcus M. Unger:** Conceptualization, Investigation, Writing - original draft, Supervision, Project administration, Funding acquisition. All authors have read and approved the final manuscript.

## Competing interests

AS is a consultant for SymbioPharm GmbH, Herborn, Germany. The other authors have declared that no competing interests exist.

## Supplementary material

Supplementary File S1 **Supplementary methods**

Supplementary Figure S1 **Alpha-diversity and beta-diversity at baseline between PD patients and controls A.** Alpha-diversity for MetaPhlAn2 data. **B.** Alpha-diversity for mOTUs2 data. **C.** Beta diversity for MetaPhlAn2 data, p 0.001. **D.** beta diversity for mOTUs2 data, p 0.001.

Supplementary Figure S2 **Intervention-associated changes in clinical scales and fecal markers A.** shows the distribution of individual scores in the CSS, NMSQ and BDI for the three study-arms prior to and post intervention. **B.** shows the distribution of fecal calprotectin concentrations in μg per g feces for the three arms prior to (baseline, white) and post intervention (8 weeks, green). For optimized scaling, seven outliers were skipped (n = 3 for PD + RS baseline; n = 2 for PD + RS 8 weeks; n = 1 for PD + DI baseline, n = 1 for PD + DI 8 weeks). **C.** shows the distribution of individual values for the percentage of fecal butyrate for the three arms prior to (baseline, white) and post intervention (8 weeks, green). Co + RS, controls plus resistant starch; PD + RS, Parkinson’s disease plus resistant starch; PD + DI, Parkinson’s disease plus dietary instructions; CSS, Constipation Scoring System; NMSQ, Non-Motor Symptoms Questionnaire; BDI, Beck Depression Inventory.

Supplementary Figure S3 **Non-metric multidimensional scaling reveals no uniform microbiome shifts associated with the intervention** Non-metric multidimensional scaling visualizing the microbiome shift associated with the intervention. As distance measure, the Bray-Curtis measure was applied. Paired data (baseline and 8 weeks) are connected with a segment. NMDS, non-metric multidimensional scaling; PD + RS, Parkinson’s disease patients receiving resistant starch; Co + RS, control subjects receiving RS; PD + DI, Parkinson’s disease patients receiving solely dietary instruction.

Supplementary Figure S4 **BusyBeeWeb Results** Annotated visualization of BusyBee Web given the contigs of a differentiated cluster. Each point corresponds to one contig derived from Parkinson’s disease patients receiving resistant starch after 8 weeks of intervention. If BusyBee assigned >50% of contigs within a cluster to the same genus, the genus annotation is provided.

Supplementary Figure S5 **Pathway Principal Component Analysis** Principal component analysis results of the center log transformed pathway annotation provided by HUMAnN2. PC, principal component; RS, resistant starch.
