# Supplementary material for: Effects of Resistant Starch on Symptoms, Fecal Markers, and Gut Microbiota in Parkinson’s Disease — The RESISTA-PD Trial
Source: Genomics Proteomics Bioinformatics. 2021 Nov 25;20(2):274–87. doi: 10.1016/j.gpb.2021.08.009 (PMC9684155; doi:10.1016/j.gpb.2021.08.009)
Supplement: Supplementary Figure S1 — Alpha-diversity and beta-diversity at baseline between PD patients and controls A. Alpha-diversity for MetaPhlAn2 data. B. Alpha-diversity for mOTUs2 data. C. Beta diversity for MetaPhlAn2 data, p 0.001. D. beta diversity for mOTUs2 data, p 0.001. [file mmc2.pdf]

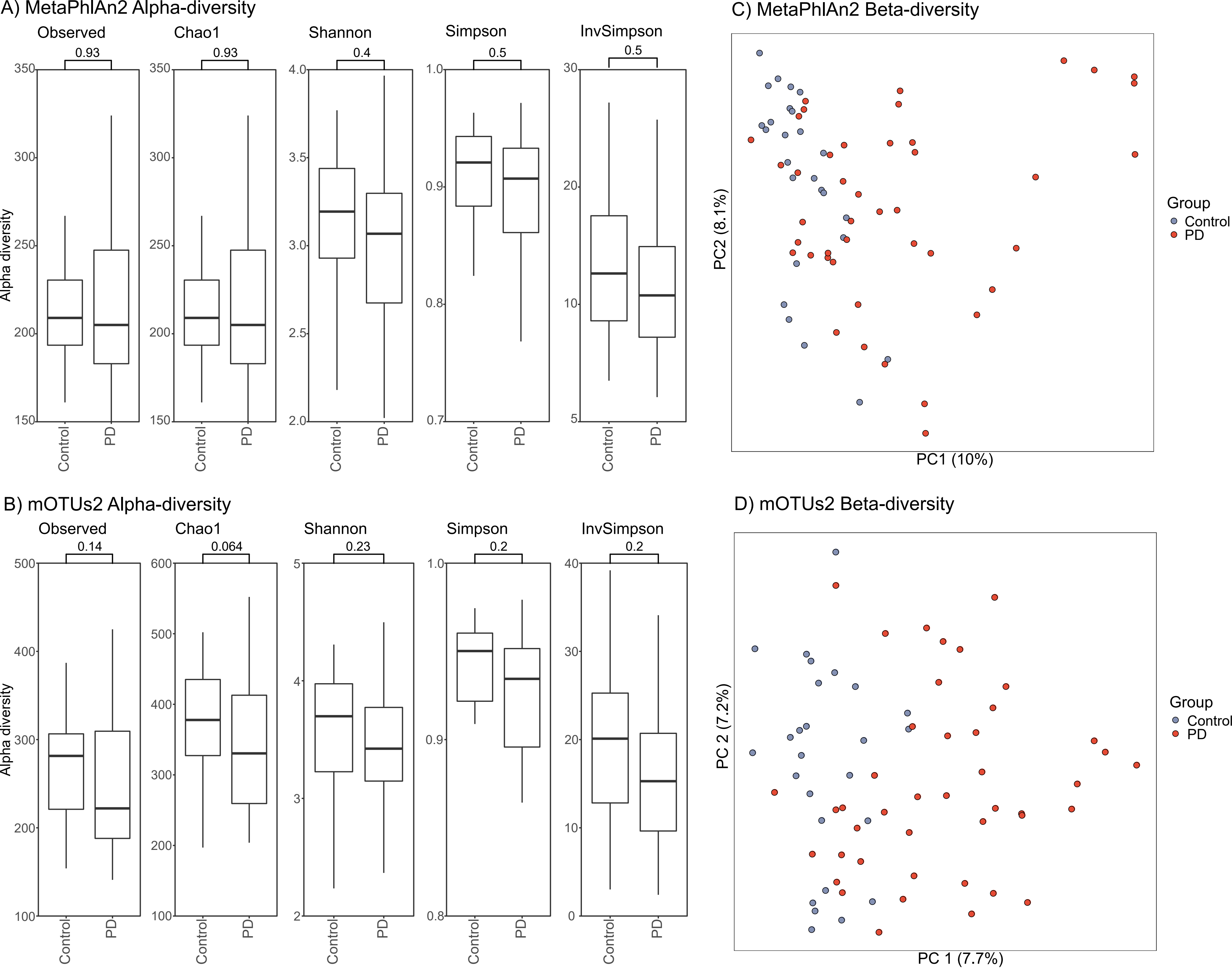
A) MetaPhlAn2 Alpha-diversity
Observed
Chao1
Shannon
Simpson
InvSimpson
0.93
0.93
0.4
0.5
0.5
Alpha diversity
Control
PD
C) MetaPhlAn2 Beta-diversity
PC2 (8.1%)
PC1 (10%)
Group
Control
PD
B) mOTUs2 Alpha-diversity
Observed
Chao1
Shannon
Simpson
InvSimpson
0.14
0.064
0.23
0.2
0.2
Alpha diversity
Control
PD
D) mOTUs2 Beta-diversity
PC 2 (7.2%)
PC 1 (7.7%)
Group
Control
PD
