# Supplementary figures and images for: Effects of Resistant Starch on Symptoms, Fecal Markers, and Gut Microbiota in Parkinson’s Disease — The RESISTA-PD Trial
Source: Genomics Proteomics Bioinformatics. 2021 Nov 25;20(2):274–87. doi: 10.1016/j.gpb.2021.08.009 (PMC9684155; doi:10.1016/j.gpb.2021.08.009)

**A**


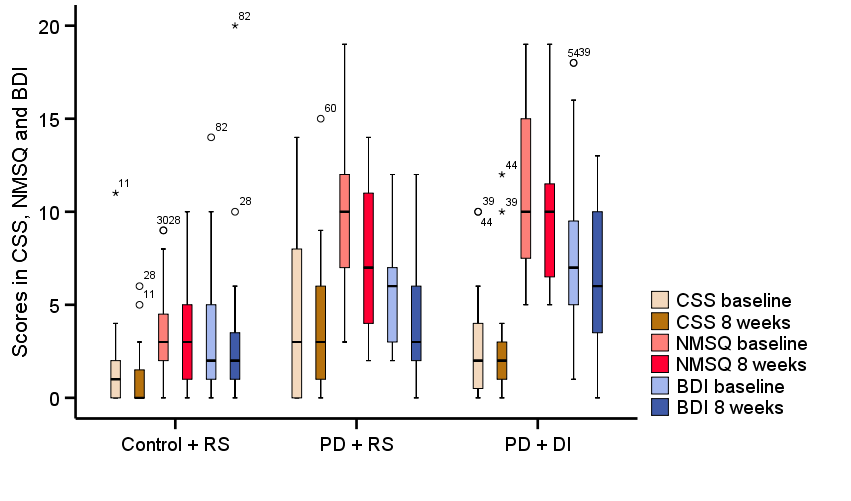


**B**


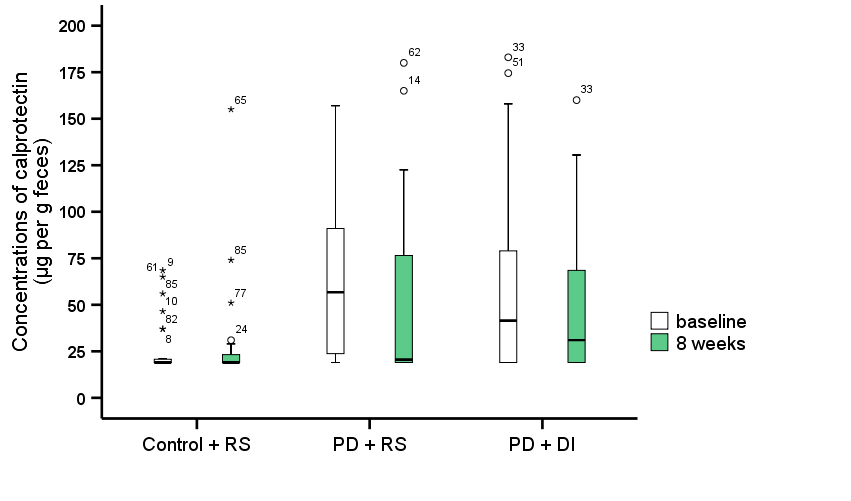


**C**


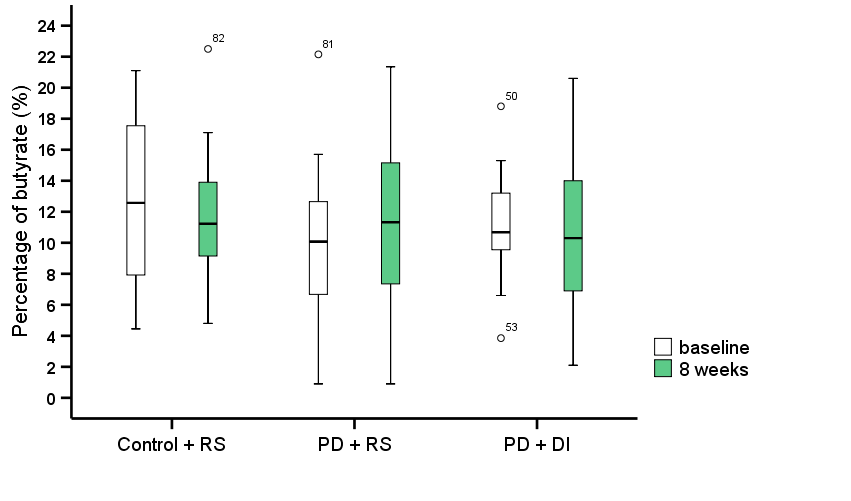

Supplement: Supplementary Figure S2 — Intervention-associated changes in clinical scales and fecal markers A. shows the distribution of individual scores in the CSS, NMSQ and BDI for the three study-arms prior to and post intervention. B. shows the distribution of fecal calprotectin concentrations in μg per g feces for the three arms prior to (baseline, white) and post intervention (8 weeks, green). For optimized scaling, seven outliers were skipped (n = 3 for PD + RS baseline; n = 2 for PD + RS 8 weeks; n = 1 for PD + DI baseline, n = 1 for PD + DI 8 weeks). C. shows the distribution of individual values for the percentage of fecal butyrate for the three arms prior to (baseline, white) and post intervention (8 weeks, green). Co + RS, controls plus resistant starch; PD + RS, Parkinson’s disease plus resistant starch; PD + DI, Parkinson’s disease plus dietary instructions; CSS, Constipation Scoring System; NMSQ, Non-Motor Symptoms Questionnaire; BDI, Beck Depression Inventory. [file mmc3.docx]

NMDS 2

NMDS 1

Group

- PD + RS
- Control + RS
- PD + DI

Timepoint

- Baseline
- ▽ 8 weeks

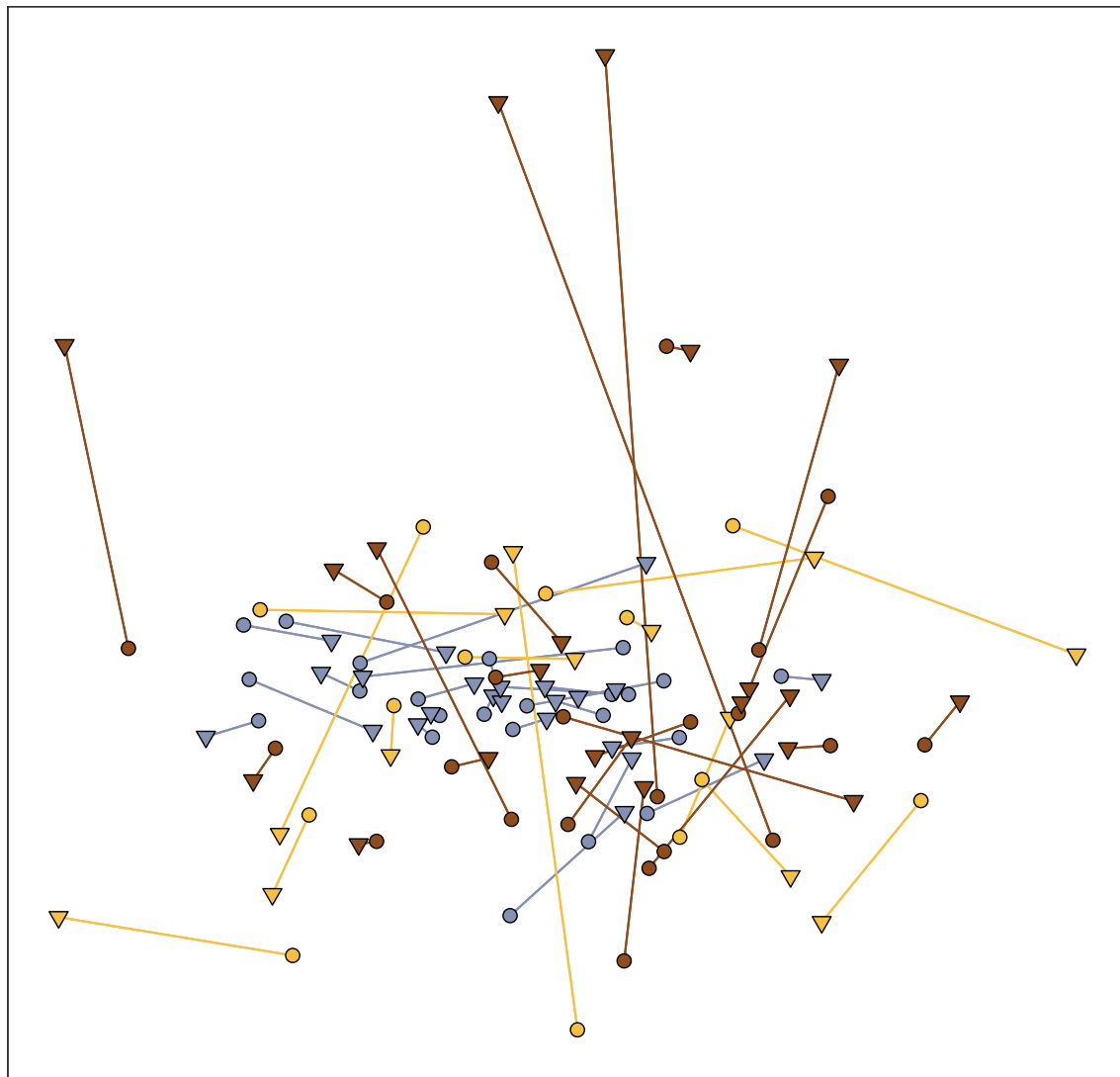

Supplement: Supplementary Figure S3 — Non-metric multidimensional scaling reveals no uniform microbiome shifts associated with the intervention Non-metric multidimensional scaling visualizing the microbiome shift associated with the intervention. As distance measure, the Bray-Curtis measure was applied. Paired data (baseline and 8 weeks) are connected with a segment. NMDS, non-metric multidimensional scaling; PD + RS, Parkinson’s disease patients receiving resistant starch; Co + RS, control subjects receiving RS; PD + DI, Parkinson’s disease patients receiving solely dietary instruction. [file mmc4.pdf]

*Propionibacterium*

*Rhodococcus*

*Rhodococcus*

*Pediococcus*

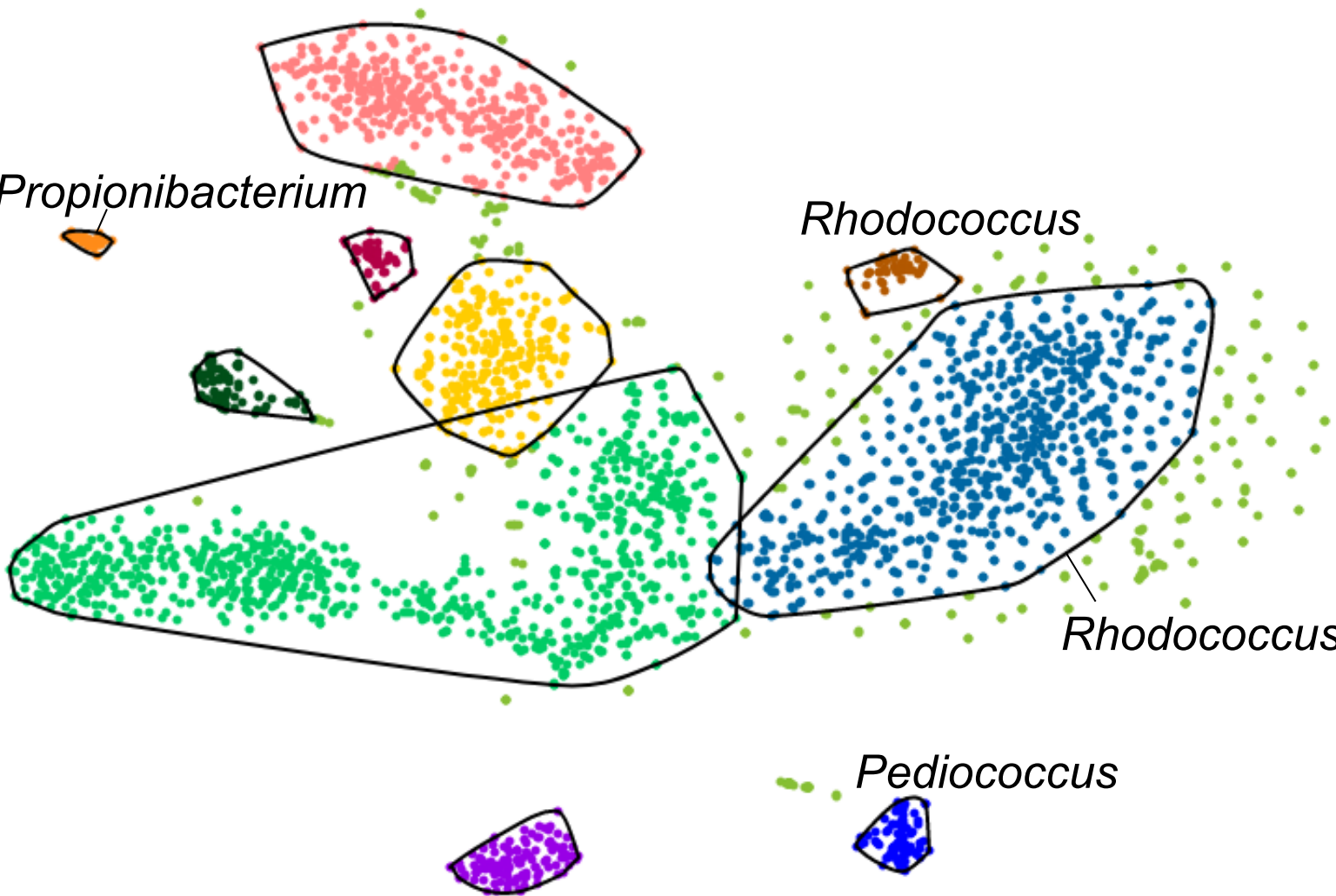

Supplement: Supplementary Figure S4 — BusyBeeWeb Results Annotated visualization of BusyBee Web given the contigs of a differentiated cluster. Each point corresponds to one contig derived from Parkinson’s disease patients receiving resistant starch after 8 weeks of intervention. If BusyBee assigned >50% of contigs within a cluster to the same genus, the genus annotation is provided. [file mmc5.pdf]

PC2 (5.9%)

PC1 (7.16%)

**Group**

- PD + RS
- CO + RS
- PD + DI

**Timepoint**

- Baseline
- △ 4 weeks
- ▽ 8 weeks

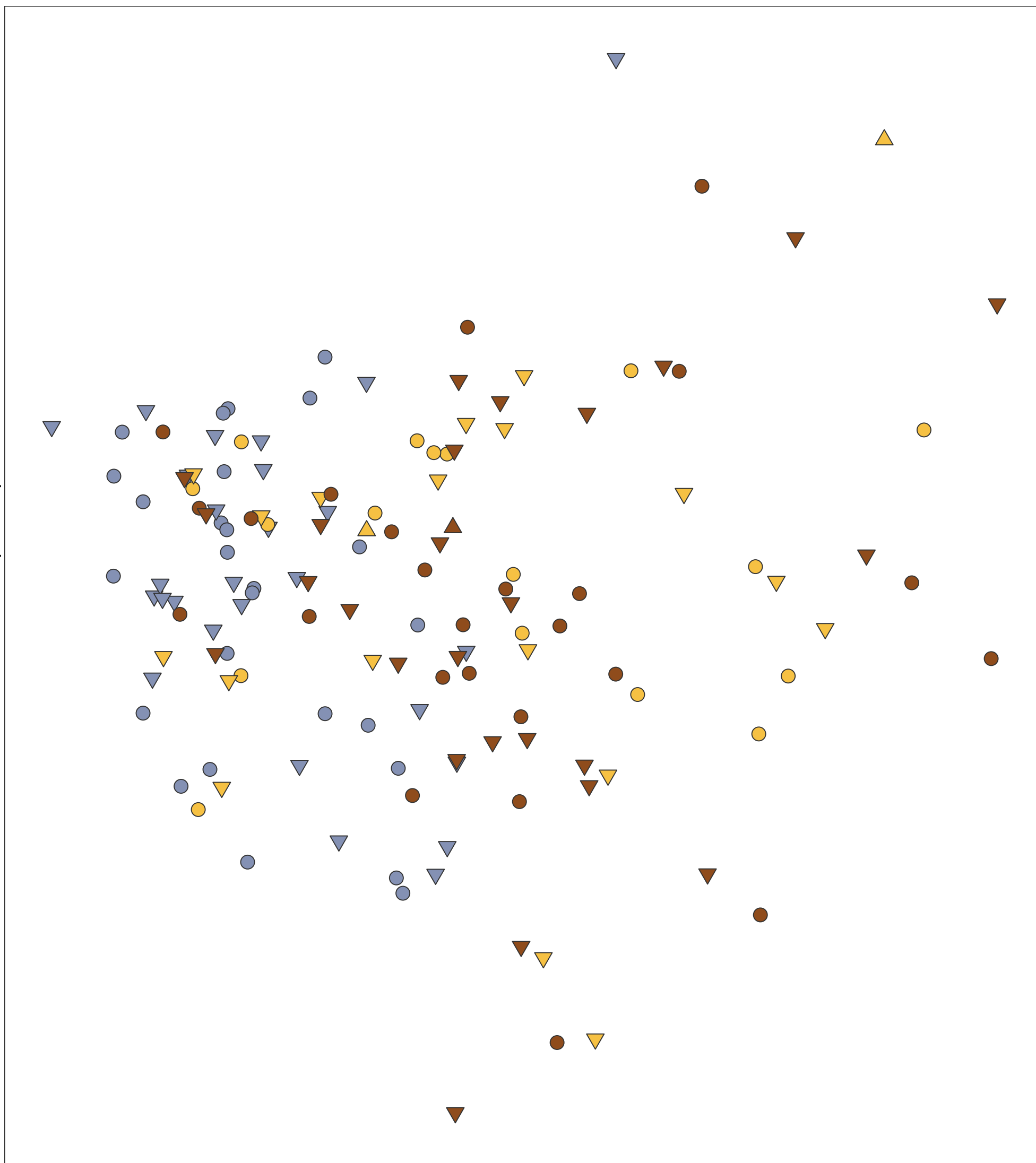

Supplement: Supplementary Figure S5 — Pathway Principal Component Analysis Principal component analysis results of the center log transformed pathway annotation provided by HUMAnN2. PC, principal component; RS, resistant starch. [file mmc6.pdf]
